# Supplementary material for: Knowledge, attitudes, and practices regarding Crimean-Congo hemorrhagic fever among general people: A cross-sectional study in Pakistan
Source: PLoS Negl Trop Dis. 2022 Dec 8;16(12):e0010988. doi: 10.1371/journal.pntd.0010988 (PMC9767344; doi:10.1371/journal.pntd.0010988)
Supplement: Attachment — Submitted filename: Responses to reviewers.docx [file pntd.0010988.s001.docx]

Dear Dr. Aysegul Taylan Ozkan,

We would like to thank you for your consideration of our manuscript titled ‘***Knowledge, attitudes, and practices regarding Crimean-Congo hemorrhagic fever among general people: A cross-sectional study in Pakistan***’ and for the valuable feedback provided. We are very grateful for the reviews provided by the editors and each of the external reviewers of this manuscript. Please find our detailed responses to comments below, in blue. All page numbers refer to the manuscript file with tracked changes. We hope the paper will now be acceptable for publication in the *PLOS Neglected Tropical Diseases.*

**Responses to Reviewer 1**

***Reviewer’s comment***: In the abstract and results sections, it is confusing to say only 51.5%, around half is not only. Please consider this.

***Authors’ response:*** Thank you for your valuable correction. Now, we have removed the term “only” with 51.5%. **[Addressed in Page No 2; Line No. 17]**

***Reviewer’s comment***: In the background section. The magnitude of the disease "CCHFV" is not clear globally or in Pakistan. Please elaborate more about the situation in Pakistan particularly after 2020 as you mentioned in discussion.

***Authors’ response:*** Dear reviewer, we have added the magnitude of "CCHFV" globally, with three more references. Also added cases report after 2020 in Pakistan with reference. **[Addressed in Page No 4; Line No. 57-61, 63,64]** **[References addressed in Page No 21; Line No. 389, 392, 397, 414]**

***Reviewer’s comment***: Culturally, I think it would be a good idea to explain what is Eid ulAdha and what is qurban with high number of animal slaughtering in a short time.

***Authors’ response:*** We have added your suggestion about Eid ul Adha. **[Addressed in Page No 4; Line No. 73-75].** More details about Eid ul Adha have already been discussed in the discussion section.

***Reviewer’s comment***: in sampling size section, I assume that the 1039 is a number of completed surveys please correct me if not and please add completeness % if possible and how did you manage missing data.

***Authors’ response:*** Yes, 1039 is a number of completed surveys and we had 100% completeness. Now, we have mentioned that incomplete submission of the survey questionnaire was not possible due to the feature in Google Forms that prevented the submission of partially answered or partially filled questions. **[Addressed in Page No 8; Line No.164-166]**

***Reviewer’s comment***: In the Questionnaire development section, please explain how did you identify cutoff points in each component (Knowledge, Attitude, Practice)? and do you think it is high in knowledge?

***Authors’ response:*** A cut-off level of ≥9 was set for categorizing the good knowledge. (Previously written ≥10 by mistake, but it was written correct in table 3 " Overall knowledge….Good (9-12)" **[Addressed in Page No7,12; Line No.141, 208, 209]**

A cut-off level of ≥34 was set for a more positive attitude.**[Addressed in Page No 8; Line No.147]**

A score of ≥19 was calibrated as good practices toward CCHF.**[Addressed in Page No 8; Line No.154]**

The knowledge section consisted of some common questions about CCHF, so it would not be difficult for participants to answer these questions. So, we considered **75%** (9 out of 12) correct answers as good knowledge.

**Similarly**, for positive attitudes (34 out of 45) and good practices (19 out of 25), we considered around **75%** as the cut-off value.

***Reviewer’s comment***: In Results section, I would like to recommend to classify the table 1 according to Have you heard about Congo fever? "in columns against other values.

***Authors’ response:*** Thanks for the suggestion. We have classified table 1. We have added the socio-demographics of those participants, who had heard about Congo fever in Table 2. We think, now it is easy to understand the analysis regarding socio-demographics.**[Addressed in Page No 10; Line No. 191]**

***Reviewer’s comment***: Also, for age ranges are very narrow, Why?

***Authors’ response:*** Dear reviewer, in the questionnaire form we had asked about age in numbers. We didn’t have option of age ranges. But in our analysis, most participants’ age ranged between 22-25 years. So, we kept the age ranges narrow.

***Reviewer’s comment***: most of participants are in urban area, does this explain low knowledge level?

***Authors’ response:*** Most of the participants in our study are from the urban area, but this does not explain low knowledge level. As shown in Table 6 (Binary logistic regression analysis to find factors of good knowledge), residence did not have significant relation to knowledge level. Although, in our study, urban population had overall good knowledge as compared to the rural population(Table 5). Also discussed briefly in the discussion section **[Addressed in Page No 17; Line No.296-302]**

***Reviewer’s comment***: in table 2, As you choose to use the questions written in the survey, please add correct response (eg: Congo fever disease was first characterized in which area? Correct "XXX")

***Authors’ response:*** We have mentioned the correct response in the table.**[Addressed in Page No 11, 12; Line No. 207]**

***Reviewer’s comment***: many questions in Attitudes and practices analysis part in not related to the same section. please clarify For example "Do you think there is a need of more awareness of CCHF in general public about CCHF" not asking about the practice.

***Authors’ response****:* There were several questions that could be categorized in either attitude section or practices section, therefore, after discussion with co-authors and our supervisor we decided to include as in the manuscript.

***Reviewer’s comment***: in the Attitudes and practices sections, questions are very directed particularly when you ask about source of knowledge and following the right procedures. (Everyone will say yes!!) my recommendation is to clarify if it is different in Urdu or to discuss this point deeply and put in limitation section. this point is very important for questionnaire validation.

***Authors’ response:*** Thanks for the comment. Yes, this point is very important for questionnaire validation. Now, we have added this point in our limitation section.

**"**A further limitation of the present study is that this study is based on self-reported data, which might contribute to social desirability. It is possible that participants may have answered attitude and practice questions positively based on what they perceive to be expected of them**"**. **[Addressed in Page No 20; Line No. 349-353]**

***Reviewer’s comment***: regarding to limitation section. I would prefer to ignore recall bias as it is very minimal in this study and consider more the limitation in the data collection tool.

***Authors’ response:*** Now, we had ignored recall bias on your suggestion. Also add further limitation regarding data collection tool. **[Addressed in Page No 19,20; Line No.344-345,347-353]**

**Responses to Reviewer 2**

***Reviewer’s comment***: The introduction section is well-defined with adequate references. The authors may include the sampling formula indicate in the online source they mentioned in methods section.

***Authors’ response:*** Dear reviewer, we have added the suggested sampling formula used in online source with its reference. **[Addressed in Page No 6; Line No.98-118]**

References (1, 2, and 3) below cite examples of recent evidence adopting similar method in sample size determination.

1. Khan FU, Khan FU, Hayat K, Chang J, Saeed A, Khan Z, Ashraf M, Rasheed UM, Atif N, Ji W, Aziz MM. Knowledge, attitude and practices among consumers toward antibiotics use and antibiotic resistance in Swat, Khyber-Pakhtunkhwa, Pakistan. Expert review of anti-infective therapy. 2020 Sep 1;18(9):937-46.
2. Campbell M. Sample size: methods of calculation and reporting. African Journal of Midwifery and Women's Health. 2019 Apr 2;13(2):1-9.
3. Abdo, Mahmoud Saeed, Mohamed Anwar Hammad, Sabariah Noor Harun, Tajunisah Mohamed Eusoff, and Siti Maisharah Sheikh Ghadzi. "Evaluation of knowledge, attitude and practice of healthcare providers towards life-threatening drug-drug interactions in Penang General Hospital, Malaysia." *Clinical Epidemiology and Global Health* (2020).

***Reviewer’s comment***: The authors should also mention why and how they divided the margins of good/poor level of knowledge with references (if there is any).

***Authors’ response:*** A cut-off level of ≥9 was set for categorizing the good knowledge. **[Addressed in Page No7; Line No. 141]**

The knowledge section consisted of some common questions about CCHF, so it would not be difficult for participants to answer these questions. So, we considered 75% (9 out of 12) correct answers as good knowledge.

***Reviewer’s comment***: The discussion sections need some incorporation of plausible explanations of the findings as well. Although the authors described its novelty, they should describe the broader implications of this study and how it may contribute to the scientific community apart from Pakistan.

***Authors’ response:*** Dear reviewer, we have added some broader implications of this study ***in discussion section.* [Addressed in Page No 19; Line No. 333-339]**

***Reviewer’s comment***: Another shortcoming of the study is its language. The author(s) may also get the manuscript revised by someone who excels in English language to enhance its quality

***Authors’ response:***

Thanks for the comment. We have tried to improve the English language.
