# Supplementary material for: Knowledge, attitudes, and practices regarding Crimean-Congo hemorrhagic fever among general people: A cross-sectional study in Pakistan
Source: PLoS Negl Trop Dis. 2022 Dec 8;16(12):e0010988. doi: 10.1371/journal.pntd.0010988 (PMC9767344; doi:10.1371/journal.pntd.0010988)
Supplement: Attachment — Submitted filename: Responses to reviewers_PNTD.docx [file pntd.0010988.s002.docx]

Dear Editor,

*PLOS Neglected Tropical Diseases*

We would like to thank you for your consideration of our manuscript ‘***Knowledge, attitudes, and practices regarding Crimean-Congo hemorrhagic fever among general people: A cross-sectional study in Pakistan***’ and for the valuable feedback provided.

We are very grateful for the reviews provided by the editors and each of the external reviewers of this manuscript. The comments are encouraging, and the reviewers appear to share our judgment that this study and its results are important. Please find our detailed response to the comments below, in blue. All page numbers refer to the manuscript file with tracked changes. We have also attached the clean files without track changes.

We hope the paper will now be acceptable for publication in the *PLOS Neglected Tropical Diseases.*

**Responses to Reviewer 1**

***Reviewer’s comment***: The author mentioned that "... and the greater the generalizability of the study"
Comment: The convenient sampling technique is not for generalization, where there is no randomization and also because of that the internet access in Pakistan was 25% of total population in 2020.
Recommendation: Delete this sentence.

***Authors’ response****: Thank you for your valuable correction. Now, we have removed the term “and the greater the generalizability”*. **[Addressed in Page No 7; Line No. 137, 138]**

***Reviewer’s comment***: The cut of points for KAP for each is not clear how it was calculated. I mean that for Knowledge why 9 not 8 or 10. and it is the same for attitude and practice scores.
Recommendation: If the author have a solid reason for that, (S)he can explain more, otherwise it will be better to use median or quartile or quantile  to describe the results.

***Authors’ response****:* *Dear Mohamed Abdelbaqy (Reviewer 1) thank you for the suggestion. We decided on the cutoff based on the recommendations of the review research committee, comprised of senior epidemiologists, doctors with relevant research backgrounds, and the authors of this manuscript. In fact, our goal is to glean more conclusive proof from the study regarding the number of people with generally sound knowledge. This line has since been added to the manuscript.*

***We used the cut-off based on the consensus of the review research committee comprised of senior epidemiologists, physicians having relevant research experiences, and authors of this manuscript.***

***Cut-off value for KAP is also used in some other articles, such as;***

1) Ferdous MZ, Islam MS, Sikder MT, Mosaddek AS, Zegarra-Valdivia JA, Gozal D. Knowledge, attitude, and practice regarding COVID-19 outbreak in Bangladesh: An online-based cross-sectional study. PloS one. 2020 Oct 9;15(10):e0239254.
2) Ahmed A, Saqlain M, Tanveer M, Tahir AH, Ud-Din F, Shinwari MI, Khan GM, Anwer N. Knowledge, attitude and perceptions about Crimean Congo Haemorrhagic Fever (CCHF) among occupationally high-risk healthcare professionals of Pakistan. BMC infectious diseases. 2021 Dec;21(1):1-9.
3) Mallhi TH, Khan YH, Tanveer N, Bukhsh A, Khan AH, Aftab RA, Khan OH, Khan TM. Awareness and knowledge of Chikungunya infection following its outbreak in Pakistan among health care students and professionals: a nationwide survey. PeerJ. 2018 Aug 30;6:e5481.

***Reviewer’s comment***: In "Statistical analysis" part the author mentioned that "Descriptive statistics were applied as means and standard deviations for continuous variables...." while in analysis there is no use of mean or SD.
Recommendation: Remove this sentence.

***Authors’ response****: Dear reviewer, we have removed that sentence. "Descriptive statistics were applied as means and standard deviations for continuous variables. "****[Addressed in Page No 10-11; Line No. 205-207]***

***Reviewer’s comment***: The results are presented very well and clear with alot of effort to get appropriate information from the data. the following are some comments to be answered in the next version.
1) the following question are not related to practice.
- Do you think keeping livestock at home poses you an extra risk of getting CCHF
- Do you think there is a need of more awareness of CCHF in general public about CCHF
- Do you think hospitals in your area are provided with sufficient facilities to diagnose and treat CCHF
Recommendation: Eighter to relocate those questions or merge the both tables (Attitude and practice) and analyse them as one component.

***Authors’ response****: Dear reviewer, thank you for the suggestion. In KAP studies generally, sometimes attitude and practice questions get overlap. These things often vary from culture to culture. As we mentioned in the methods, we developed the questionnaire in consultation with the research committee (comprised of experts) and did all questionnaire development steps to ensure that it is well validated and will be able best to reflect the attitude and perceptions of the Pakistani population. Therefore, we are hopeful that you will understand our point of view.*

***Reviewer’s comment***: **Just small comment** "Attitudes and practices" section, after table 4; the author mentioned that "The majority of participants 40 .9% (n=219) strongly agreed that there...  ", actually we cannot say majority where the percentage is 40%. Please review

***Authors’ response****: Correction is done. Now, we have removed the term “The majority of* participants”.**[Addressed in Page No14; Line No. 250]**

**Responses to Reviewer 2**

The paper is written well and presents some interesting insights on a relatively neglected field of knowledge. The background section is well-defined with adequate references. However, the authors should mention why they chose convenient sampling method when the study was conducted on general population. Although they mentioned they used digital platforms which was more accessed by graduate level people in the limitations, they referred to further studies instead for providing justification for how they addressed the shortcomings. Apart from that, the results and the discussion sections are described properly. I encourage the authors to address these minor issues, and wish them all the best. Thank you.

***Authors’ response****:* *We adopted convenient sampling because it is extremely speedy, easy, readily available, and cost-effective. Although there are biases in this sampling technique, we covered them by taking a larger sample size and inviting participants from all types of general populations. We have also included the study's limitations so that readers and policymakers will understand its shortcomings.*

*Changes done in methods Study design and setting*
