# Supplementary material for: Knowledge, attitudes, and practices regarding Crimean-Congo hemorrhagic fever among general people: A cross-sectional study in Pakistan
Source: PLoS Negl Trop Dis. 2022 Dec 8;16(12):e0010988. doi: 10.1371/journal.pntd.0010988 (PMC9767344; doi:10.1371/journal.pntd.0010988)
Supplement: Attachment — Submitted filename: Responses to reviewer comments.docx [file pntd.0010988.s003.docx]

Dear Editor,

*PLOS Neglected Tropical Diseases*

We would like to thank you for considering our manuscript ‘***Knowledge, attitudes, and practices regarding Crimean-Congo hemorrhagic fever among general people: A cross-sectional study in Pakistan***’ and for the valuable feedback provided.

We are very grateful for the reviews provided by the editors and each of the external reviewers of this manuscript. The comments are encouraging, and the reviewers appear to share our judgment that this study and its results are important. Please find our detailed response to the comments below in blue. All page numbers refer to the manuscript file with tracked changes. We have also attached the clean files without track changes.

We hope the paper will now be acceptable for publication in the *PLOS Neglected Tropical Diseases.*

**Responses to Reviewer 1**

***Reviewer’s comment***: I would like to thank the authors for their valuable work and scientific discussion they have with this paper, also for clarifying the previous comments.  A few points would like to discuss as following
- Most of the previous points were discussed clearly.
- Regarding the "cut-off points", what you mentioned as a response for the previous review round sounds very clear. The point is to share with readers of the journals. So, you can explain how the review research committee decide that and how you follow the recommendations of review research committee.
- Regarding the "Attitude and practice" part, it is totally understandable the point of culture variation and the translation from language to another. Yes some verbs can give behaviour meaning in language and attitude meaning in another language. but here the situation is totally different, the language of manuscript is English and the reader might not get it, please be sure that there is no overlap between attitude and practice in the manuscript.
Fo example: the following questions you mentioned in manuscript
          1- Do you think keeping livestock at home poses you an extra risk of getting CCHF
          2- Do you think there is a need of more awareness of CCHF in general public about CCHF
          3- Do you think hospitals in your area are provided with sufficient facilities to diagnose and treat CCHF
how you can clarify "do you think" to action??!!
As a recommendation please merge the attitude and practice as one part, OR, review the translation of "do you think" in Urdu is a practice verb, Or remove this part, and of course reflect to regression

***Authors’ response****:* Dear reviewer, we are very thankful for your valuable suggestions and we acknowledge your recommendation. Now, we have reviewed these questions and made some corrections. For example, following questions;

1) Do you think if someone in your family gets CCHF, he/she should be admitted to a hospital or other healthcare facility (Attitude). 2) There is a need of more awareness of CCHF in general public about CCHF (Practice). 3) Keeping livestock at home poses you an extra risk of getting CCHF (Practice). 4) Hospitals in your area are provided with sufficient facilities to diagnose and treat CCHF (Practice). **[Addressed in Page No 14,15; Table No. 04,05]**

**Responses to Reviewer 2**

***Reviewer’s comment***: The methods section looks well-described. The objectives of the study is clearly articulated, and the authors used appropriate study design. The sample size, hypothesis test and the analysis done are appropriate. However, the authors should mention how they constructed the questionnaire. They should provide references if they used any, or just state if they constructed an inventory on their own. Providing references would strengthen how they labeled any knowledge poor or an attitude positive/negative.

***Authors’ response****:* Dear reviewer, construction of the questionnaire is well explained in the questionnaire development section. References of literature (references number 2, 3, 28**)** for questionnaire developments are also provided in that section. **[Addressed in Page No 08; Line No. 148-149]**

***Reviewer’s comment***: The results clearly and completely presented. The analysis presented also look standard

***Authors’ response****:* Dear reviewer, Thank you for your appreciation.

***Reviewer’s comment***: The authors may consider adding a few sentences of recommendations based on the specific findings from binary logistic regression analysis. For example, what are the implications of the association found between education and income status and the KAP of the participants. More specifically, indicate what needs to be done.

***Authors’ response****:* The mentioned deficiency had been included in the conclusion section of the manuscript.**[Addressed in Page No 21; Line No. 384-386]**
